# Placental Molecular Expression of Different Pathogenic Vaginal Infections

**DOI:** 10.3390/ijms26072863

**Published:** 2025-03-21

**Authors:** Daniela Roxana Matasariu, Constantin Condac, Victoria Bîrluțiu, Ludmila Lozneanu, Iuliana Elena Bujor, Vasile Lucian Boiculese, Mihai Sava, Alexandra Ursache

**Affiliations:** 1 Department of Obstetrics and Gynecology, “Cuza Voda” Hospital, 700038 Iasi, Romania; daniela.matasariu@umfiasi.ro; 2 Department of Mother and Child, University of Medicine and Pharmacy “Gr. T. Popa”, 700115 Iasi, Romania; iuliana-elena.bujor@d.umfiasi.ro (I.E.B.); alexandra.ursache@umfiasi.ro (A.U.); 3 Department of Anesthesia and Intensive Care, “Cuza Voda” Hospital, 700038 Iasi, Romania; costicondac@gmail.com; 4 Department of Infectious Diseases, University of Medicine and Pharmacy “Lucian Blaga”, 550169 Sibiu, Romania; victoria.birlutiu@ulbsibiu.ro; 5 Department of Morpho-Functional Sciences I—Histology, University of Medicine and Pharmacy “Gr. T. Popa”, 700115 Iasi, Romania; 6 Biostatistics, Department of Preventive Medicine and Interdisciplinarity, University of Medicine and Pharmacy “Gr. T. Popa”, 700115 Iasi, Romania; lboiculese@gmail.com; 7 Department of Anesthesia and Intensive Care, University of Medicine and Pharmacy “Lucian Blaga”, 550169 Sibiu, Romania; mihai.sava@ulbsibiu.ro

**Keywords:** vitamin D receptor, CD44, osteopontin, COX-2, placenta, Klebsiella, Group B Streptococcus, Ureaplasma urealyticum, Candida, chorioamnionitis

## Abstract

This study evaluated the differential expression of four placental markers—vitamin D receptor (VDR), Cluster of Differentiation 44 (CD44), osteopontin (OPN), and cyclooxygenase-2 (COX-2)—in response to pathogens, which may contribute to our understanding of pathogen-specific impacts on pregnancy outcomes. We immunohistochemically (IHC) analyzed placental tissues obtained from 70 healthy-term pregnant women in the control group and compared them to tissues obtained from 78 women with pregnancy above 24 weeks of gestation, single-pathogen vaginal infection, and premature rupture of membranes/preterm premature rupture of membranes (PROM/PPROM). We detected high expression of these four molecules in cases of *Group B Streptococcus (GBS)* and *Ureaplasma urealyticum* vaginal infections, and moderate expression in cases of *Enterobacteriaceae* infections, except for *Klebsiella*; the cases with *Klebsiella* and *Candida species* (spp.) vaginitis exhibited a lower expression compared to the healthy control group. VDR, CD44, and OPN had increased placental expression in *GBS* and *Ureaplasma urealyticum* vaginal infections; the opportunistic pathogenicity of both *Escherichia coli* and *Candida* spp. explains their low IHC positivity, and the tremendous ability of Gram-negative bacteria to elude the host immunity is revealed by the negative IHC staining in cases of *Klebsiella* vaginitis. These findings suggest that pathogen-specific alterations in the expression of these markers may contribute to the differential risk stratification of pregnancy complications and may mitigate the risks of adverse maternal and fetal outcomes. Interventions aiming to modulate these pathways might improve pregnancy outcomes.

## 1. Introduction

One of the most frequent pathologies encountered during pregnancy is vaginal infections [1], which play a significant role in adverse maternal and fetal outcomes, increasing both neonatal and maternal morbidity and mortality [2]. Vaginal infections can lead to endometritis, preterm birth (PB), premature or preterm premature rupture of membranes (PROM/PPROM), abortion, intrauterine growth restriction (IUGR), low birth weight (LBW), fetal infection, and, in severe cases, fetal demise [2,3,4]. Once the infection reaches the amniotic sac, it may result in histological or clinical chorioamnionitis, with or without PROM/PPROM [5,6,7]. Chorioamnionitis is frequently recognized as having a polymicrobial etiology [8]. In some cases, infections trigger sterile intra-amniotic inflammation, which alone can result in PB [9].

The existing studies underline our limitations in identifying the infectious causes for intra-amniotic inflammation, with estimates suggesting the prevalence is likely underestimated. The prevalence seems to range between 13% and 48% in women with intact or ruptured membranes and PB, and between 46% and 53% in women with clinical and/or histological chorioamnionitis [10,11,12]. Studies have underlined that the immune response in amniotic fluid varies from pro-inflammatory to anti-inflammatory, a phenomenon that remains incompletely comprehended and necessitates further research [7].

A variety of micro-organisms are implicated in women’s genital tract infections during pregnancy, including *Group B Streptococcus* (*GBS*), *Escherichia coli* (*E. Coli*), *Neisseria gonorrhoeae*, *Enterococcus faecalis*, *Enterobacter* spp., *Trichomonas vaginalis*, *Candida* spp., and *bacterial vaginosis* (*BV*). Microbes responsible for pelvic inflammatory disease, such as *Ureaplasma urealyticum*, *Mycoplasma hominis*, *and Chlamydia trachomatis*, also contribute [2]. Notably, *Mycoplasma and Ureaplasma* produce cytotoxic substances that amplify inflammation by inducing cyclooxygenase-2 (COX-2) synthesis and subsequent prostaglandin formation [2]. Hormonal changes during pregnancy increase susceptibility to *Candida* spp., with vaginal colonization observed in approximately 40% of pregnant women, although it generally results in less severe complications compared to other infections, such as late miscarriage, PB, and chorioamnionitis [2]. To uncover part of this puzzle, we conducted this research evaluating four closely linked molecules with significant roles in inflammation, placental structure, and function: vitamin D receptor (VDR), Cluster of Differentiation 44 (CD44), osteopontin (OPN), and cyclooxygenase-2 (COX-2).

VDR mediates the endocrine, paracrine, and autocrine actions of 1,25 dihydroxy vitamin D [13,14]. While VDR is primarily involved in calcium metabolism, it also exerts a multitude of other effects, among which the anti-inflammatory and immune modulatory effects are of high importance [14]. The suppression of tumoral necrosis factor α (TNF-α) and COX-2 is the mechanism through which vitamin D manages to reduce inflammation in placental trophoblast. VDR is extensively expressed in the placenta during pregnancy [15].

CD44 is a frequently encountered surface molecule with essential roles in adhesion and cell-to-cell and cell-to-matrix interactions. It has many extracellular ligands, such as hyaluronic acid (HA). CD44 exhibits an interesting in vitro role in lymphocyte-to-venule adhesion and in cytokine macrophage production, being highly expressed in the placental tissue starting from the second trimester of pregnancy according to the literature, as well as being supposedly involved in the limitation of trophoblast invasion [16,17,18,19]. In 2019, Mahendroo et al. investigated the expression of CD44 and HA in the cervical epithelia of mice during pregnancy infections. Their research suggested that HA depletion at the cervical epithelial level disrupts the natural barriers, allowing micro-organisms to ascend into the upper genital tract and cause preterm birth [16].

Another adhesion and cell invasion extracellular matrix marker that influences and modulates cell function by interacting with CD44 and other ligands is OPN [20,21,22]. It seems to also be a key participant in the development of the placenta and exhibits altered expression patterns in many pregnancy-related pathologies [23,24,25,26,27]. Animal studies depicted NK cells as the main source of OPN, which, despite being part of the extracellular matrix, also functions as a cytokine [22,28,29,30].

COX-2 isoform, a controlling factor in the production of prostaglandins, is expressed in only a limited variety of tissues, including the placenta [31]. The study by Cao et al. in 2021 showed that vitamin D determines the downregulation of COX-2 expression in the placenta, thus reducing inflammation. Since COX-2 is responsible for producing inflammatory molecules, its reduction helps mitigate placental inflammation [15].

Vitamin D seems to be VDR-dependent and directly modulates both the innate and adaptive immune responses [32]. Most studies have underlined the downregulation of CD44 expression via the vitamin D–VDR complex [33,34]. In addition, elevated vitamin D levels determine COX-2 downregulation [15]. Paracrine and autocrine signaling of the CD44-OPN complex promotes extravascular cell migration to inflammation sites, with OPN enhancing the expression of CD44 on the surface of various cell types [33,34]. We chose to study these highly interconnected markers within the context of inflammation because of their significant impact on the immune response pathways.

This research aimed to investigate how different types of vaginal infections during pregnancy alter the expression of VDR, CD44, OPN, and COX-2, thereby contributing to structural and functional changes in the placenta. All of these molecules seem to contribute to varying degrees in the physiopathology of preterm birth, with its consequent unfavorable maternal and fetal outcomes. Through analyzing these markers, we hope to obtain a plausible explanation as to why some micro-organisms have a more significant negative impact on maternal and fetal well-being than others.

## 2. Results

We included 70 healthy-term pregnant women in the control group and 78 women with pregnancy over 24 weeks of gestation and with single-pathogen vaginal infections. In our analysis, we detected statistically significant differences in gestational age at birth. Pregnant women without vaginal infections, on average, delivered 5 weeks later than those from the group with vaginal infections. As a consequence, the average birth weight between the two groups also differed by approximately 1000 g. As expected, the average CRP value was also statistically significantly 8 times higher in pregnant women with vaginal infections (Table 1).

The results obtained show a multitude of pathogens involved in vaginal infections, with *E. coli* and *GBS* occupying the first two positions, followed closely by *Candida* spp., *Enterococcus*, and *Ureaplasma urealyticum*, with almost similar percentages (Table 2). The lowest incidence pertained to *Klebsiella vaginal* infection (Table 2).

The univariate analyses of our markers indicated variability in their expression, with certain infections showing distinct patterns that could influence the inflammatory and immune responses in the placenta during pregnancy (Table 2 and Table 3). Pregnant women with vaginal infections had lower levels of CD44 compared to the controls, but the difference did not reach statistical significance, with a *p*-value of 0.185. OPN showed different expression levels depending on the pathogen involved, with statistically significant higher expression in the vaginal infection group compared to the controls, with a *p*-value of 0.004 (OR 2.93). COX-2 was expressed in nearly all cases, without any statistically significant difference between the two groups. VDR was found to be positive in three-quarters of the vaginal infection cases, without significant difference between the two groups, with a *p*-value of 0.907 (Table 4). Our results suggest that pregnant women have a 1.67 increased risk of experiencing a vaginal infection if they are CD44-negative, a 1.102 increased risk of experiencing a vaginal infection if they are VDR-negative, and a 2.933 increased risk of having a vaginal infection if they are OPN-positive (Table 4).

**Figure 1:**
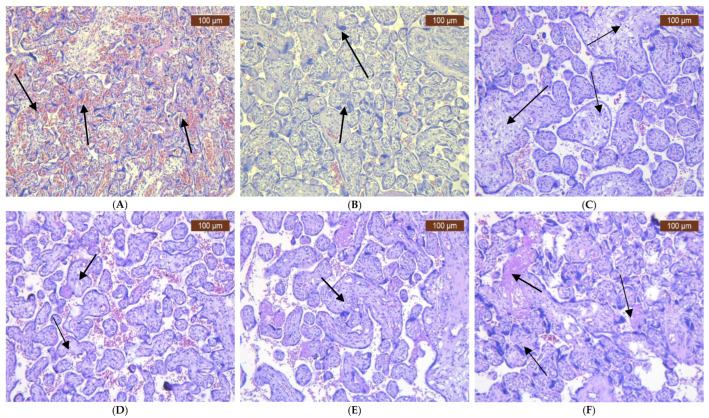
Histopathological changes in the placenta from women with vaginal infection. (**A**) *E. coli* infection. Areas of hemorrhage in the villous and intervillous space (black arrows) (HE×10). (**B**) *Klebsiella* infection—cytotrophoblast layer showing large, irregular, and hyperchromatic nuclei with more eosinophilic or amphophilic cytoplasm (black arrows) (HE×10). (**C**) *GBS* infection—the villous space is loose and edematous, with many fetal blood vessels and rare cells (black arrows) (HE×20). (**D**) *Enterococcus faecalis* infection—the villous space is dens with less vessels and rare cells (black arrows) (HE×10). (**E**) *Candida* spp. Infection—membrane infiltration (black arrows) (HE×10). (**F**) *Ureaplasma urealyticum* infection—placental fibrinoid and lymphocytic infiltration (black arrow) (black arrows) (HE×10).

The histological characteristics of the placenta varied according to the infections caused by the isolated pathogens (Figure 1A–F). In *E. coli* infections, there was damage to the placental villi, including areas of necrosis, significant inflammation, and occasional thrombi within the chorionic vessels (Figure 1A). *Klebsiella* infections also showed destruction of the chorionic villi, accompanied by fibrosis and more active and intensive inflammatory response (Figure 1B). *GBS* infections led to inflammatory infiltrates of neutrophils in the fetal membranes (amnion and chorion), fetal blood vessels within the chorionic plate, stem villi, and chorionic villi (Figure 1C). The presence of *Enterococcus* faecalis resulted in membrane infiltration and occasionally affected the villi, with small clots in the placental vessels, which impaired blood flow (Figure 1D). *Candida* spp. infections were characterized by noticeable lymphocytic infiltration (Figure 1E). In *Ureaplasma urealyticum* infections, there was less severe neutrophilic infiltration of the membranes, along with prominent lymphocytic infiltration, syncytial knots, and intervillous fibrin (Figure 1F); in some cases, small thrombi in the chorionic vessels were observed, resulting from extensive inflammation and vascular injury.

**Figure 2:**
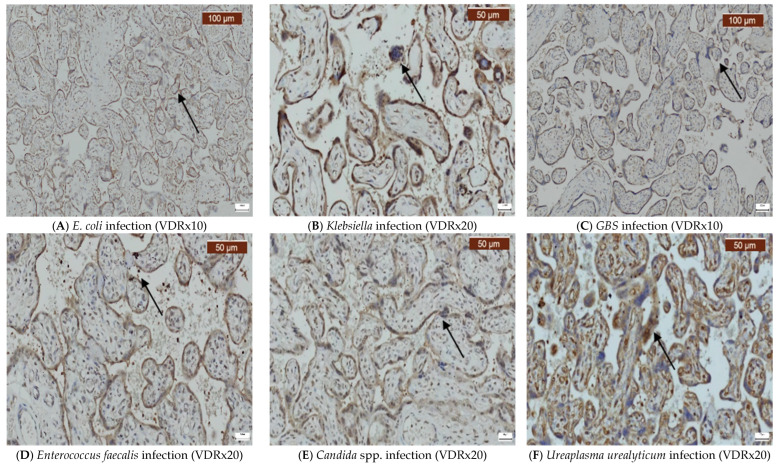
Immunohistochemical placenta expression of the VDR. The arrows indicate positive staining.

**Figure 3:**
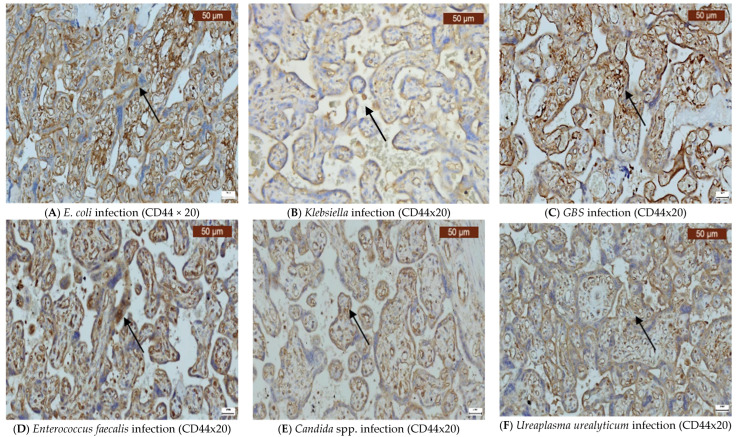
Immunohistochemical visualization of the CD44 in the plasma membrane of some extravillous cytotrophoblastic cells in the basal plate, in the mesenchymal cells (histiocytic component), and the large majority of the decidual cells showed positive immunostaining for this marker. The arrows indicate positive staining.

**Figure 4:**
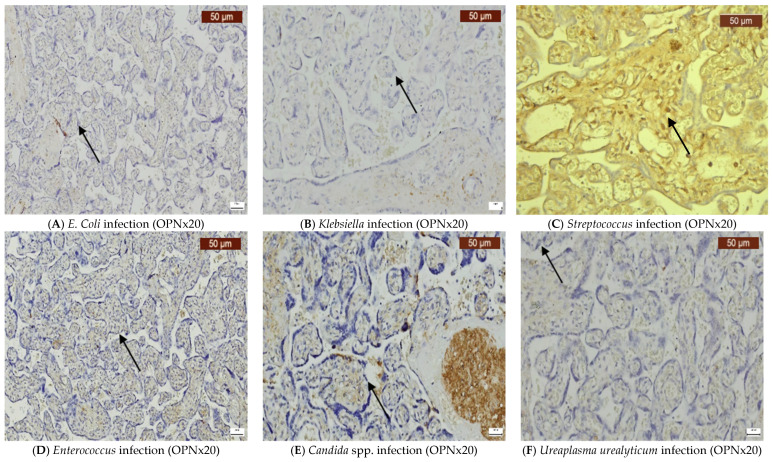
OPN staining was absent or very weak, being noticed at the periphery of chorionic villi (cytotrophoblast and syncytiotrophoblast layers on the surface of villi). The arrows indicate positive staining.

**Figure 5:**
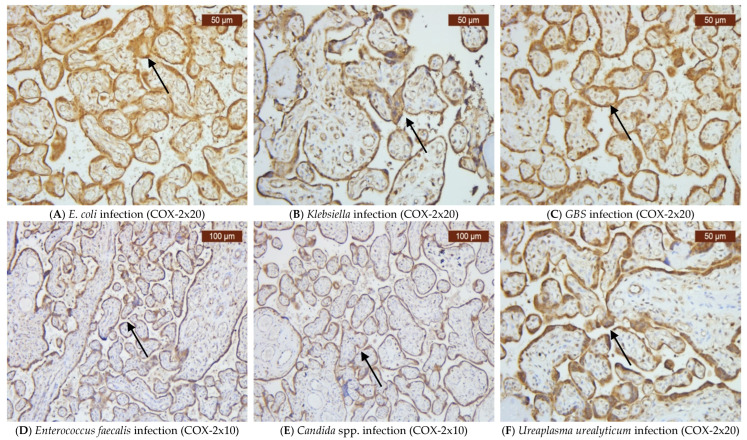
COX-2 was primarily expressed in the various villous types, especially in stem villi, amniotic epithelial cells, chorionic trophoblasts, decidual cells, and weak staining in the mesenchymal cells. The arrows indicate positive staining.

**Figure 6:**
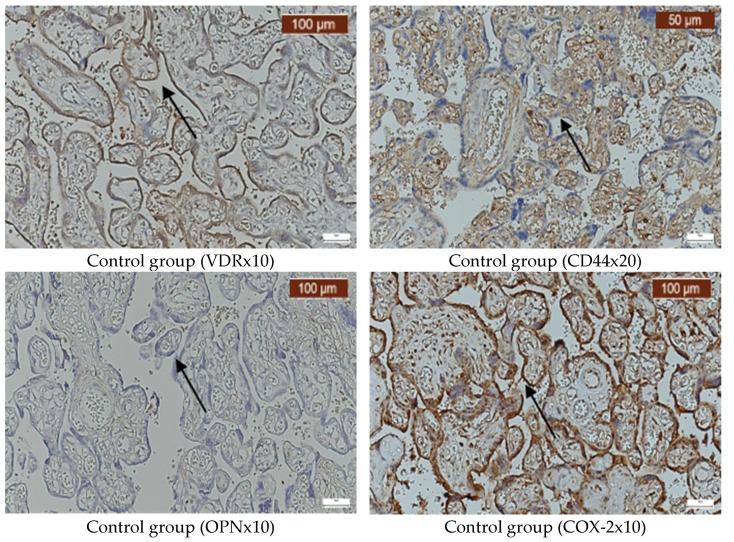
VDR, CD44, OPN, and COX-2 immunohistochemistry analysis from the control group. The arrows indicate positive staining.

Infections caused by *E. coli*, *Klebsiella*, *GBS*, *Enterococcus faecalis*, *Candida* spp., and *Ureaplasma urealyticum* can significantly alter the expression of VDR, CD44, OPN, and COX-2 in the placenta. Our findings indicate that VDR expression was generally variable, but it often decreased (Figure 2A–F), while the expression of CD44 was elevated in most infections (Figure 3A–F). OPN was largely absent (Figure 4A–F), while, in contrast, COX-2 expression was elevated in the majority of cases with vaginal infections (Figure 5A–F). The immunohistochemical analysis of the control group is illustrated in Figure 6.

## 3. Discussion

Many pieces are missing from the puzzle of maternal and fetal outcomes in pregnant women with different vaginal infections. Oh et al. underlined our limitations in identifying infections through standard vaginal culture techniques in women with preterm birth and clinical chorioamnionitis [33]. Some studies in the literature reveal a high prevalence of bacterial vaginosis in women experiencing premature birth and chorioamnionitis [2,34]. Despite a low incidence of septicemia caused by these germs, placental and fetal cultures show a diverse range of pathogens. Additionally, there are commensal germs in the vaginal flora that can sometimes exhibit pathogenic action (depending on the time frame of colonization, differences in strain virulence, interactions with other germs, and/or suppression of the maternal immune response) and negatively impact the evolution of pregnancy [2]. One such example is *Ureaplasma* spp., which has a high colonization rate but is not always associated with inflammation and infection [2]. The accuracy of vaginal cultures in identifying the micro-organisms responsible for the infection improves as the gestational age decreases in cases of chorioamnionitis [35]. Despite the fact that there is a considerable number of cases in which clinical signs of infections are missing, more than half of these women have histological signs of chorioamnionitis with positive cultures [35].

Our study examined the controversy regarding these aspects in the literature, hoping to offer a plausible histological explanation for the existing inconsistencies. Due to the fact that the vaginal microbiome is extremely diverse, depending on a multitude of extrinsic and intrinsic factors, we decided to study placental key surface molecules to detect the actual impact of vaginal infections in the placenta. The clinical manifestations of the women with vaginal infections included in our study varied a lot, ranging from patients with characteristic signs and symptoms of chorioamnionitis to asymptomatic patients.

Similar to our observations, the studies by Al-Adnani et al. and N. J. Sebire et al. revealed septic infarcts, intervillous thrombi, and necrotizing villitis accompanied by perivillous inflammation in women with vaginal infections, in contrast to the findings from the control group in the placental H&E examination [8,36].

In accordance with the literature results, our results display a lower gestational age with consequent lower birth weight in women with PROM or PPROM and positive vaginal cultures [37,38,39]. The vaginal infection group exhibited a slightly lower Apgar score compared to the controls. The minimal difference may be attributed to our inclusion criteria, which selected only pregnancies > 24 weeks of gestation with vaginal positive cultures, and term pregnancies with negative vaginal cultures for the control group. The group with vaginal infections exhibited a higher leucocyte count compared to the control group. However, the difference was not statistically significant. A possible explanation could be that these women arrived at the hospital under 12 h after PROM/PPROM. During this short 12-hour time frame, leukocyte mobilization might have begun but had not yet reached a sufficient level to consistently differentiate from the control group. Additionally, upon admission, these patients were promptly administered empiric antimicrobial therapy, which could also impact this response. After admission, vaginal cultures and blood samples were collected. The difference in CRP values between the two groups was statistically significant, with higher levels observed in the vaginal infection group.

The placenta is essential for fetal development and well-being. Notwithstanding extensive research on its multitude of roles and functions, many aspects remain largely unknown [15]. The four molecules examined in this study—VDR, CD44, OPN, and COX-2—are involved in many placental processes and recognize an ascending trend depending on the gestational age during pregnancy [15,16,17,18,19,22]. Our results reveal that *GBS* and *Ureaplasma urealyticum* exert the most significant impact on the four studied markers, leading to high levels of VDR, CD44, OPN, and COX-2 in the placental tissues. The presence of *Enterococcus* and the absence of vaginal infections in the control group exhibit almost the same impact on the levels of the above-mentioned markers, with OPN being the least affected. Infection by *Escherichia coli* leads to a higher positive percentage of these markers in the patients with vaginal infections than the control group, while infection by *Candida* spp. results in minor alterations in the tissue expression of these markers, being lower than the expression levels observed in the control group. The least significant impact is exerted by *Klebsiella* infection. Therefore, according to our observation, the most aggressive micro-organisms remain to be *GBS* and *Ureaplasma*.

*GBS* is the most commonly identified perinatal germ, with guidelines establishing the time frame for its screening during the third trimester, along with therapeutic and prophylaxis treatment. The unfavorable maternal and neonatal outcomes resulted from *GBS* infection have been long acknowledged [40]. Its virulence also depends on the presence of adhesive and invasive molecules, and its ascension toward the uterine cavity is ensured by the destruction of hyaluronic acid (HA) through its hyaluronidase production [41]. The CD44 adhesion molecule plays a significant role in placental development, particularly through its involvement in angiogenesis. Its primary ligand, HA, contributes to maintaining the structural integrity of the placenta by supporting extracellular matrix organization and cellular interactions [42]. An interesting 2017 review by Jordan et al. underlines that transgenic mice with low CD44 expression prove to be protected from *Group A Streptococcus* infection [43]. CD44 expression is upregulated in infections that modulate local immune response. Thus, our results align with the literature findings regarding the major negative impact of *GBS* infection and its consequences in pregnancy. Another CD44 ligand, with known implications in angiogenesis and in the modulation of the immune response, is OPN [44,45]. The study by Diao et al. (2011) suggests that OPN expression is rapidly elevated in response to an infection [46], and this increase appears to be associated with the severity of the infection [47]. As depicted in our study as well, OPN levels seem to correlate to an extent with CRP values and increase in pregnant women with PROM. The lack of a complete correlation may be linked to the fact that, unlike CRP, which is a systemic inflammatory marker whose levels tend to be influenced by any infection, regardless of the pathogen’s virulence, high OPN levels might be more specific for severe immune responses, while its levels remain unaltered with opportunistic infections [48].

Even if the literature results reach unanimity regarding the causative link between *GBS* vaginal infection and unfavorable pregnancy outcomes, research should still endeavor to examine the consequences of *Ureaplasma urealyticum* vaginal infection. The existing results have failed in placing this germ into one of the two categories of being a simple commensal germ to being an extremely virulent one leading to chorioamnionitis [49]. Although this incertitude persists in the literature, our results are similar to those described by de Oliveira et al. in 2020 [50]. *Ureaplasma urealyticum* virginal infection seems to trigger significant placenta effects. Thus, all three structural placental molecules examined in this study, which are involved in cell-to-cell and cell-to-matrix adhesion, cellular migration and differentiation, angiogenesis, and modulation of the local immune response, have severely altered expression in *GBS* and *Ureaplasma urealyticum* virginal infections [20,44,45].

The tissue samples from all the pregnant women in this study, including those with vaginal infections and the control group, exhibited similarly intense positivity for COX-2. Thus, based on our study findings, this molecule’s expression is more linked to inflammation rather than being infection-specific. The high levels observed in both groups can be explained by the fact that we collected placenta tissues from the control group whose pregnancies were close to the time of labor, showing increased COX-2 expression, with consequent prostaglandin production that initiated birth [51].

Vitamin D, mediated by VDR, exerts a key role in reproductive tissues, including the vagina. It regulates antimicrobial molecules that protect against bacterial infections and may also influence defensin production and neutrophil function [50,52]. Bespalova et al. made an interesting observation regarding vitamin D supplementation, with the consequent favorable result of decreasing the frequency of *E. coli* vaginal infection [53]. Furthermore, as suggested by Cao et al. (2021), vitamin D downregulates COX-2 signaling and expression, thus reducing inflammation [15]. We identified increased placental VDR levels in cases with *SGB*, *Ureaplasma urealyticum*, and *Enterococcus* vaginal infections. Vaginal yeast infection is frequently encountered in pregnancy, especially due to hormonal changes that predispose women to develop such pathology [54]. Despite being a commonly encountered vaginal infection pathogen, infection by *Candida* spp. rarely leads to chorioamnionitis [55], although there are studies that describe placental structural changes in some cases [8]. The tissue samples of the vaginal infection-free control group exhibited similar VDR expression levels to women with *Candida* spp. and *E. coli* vaginitis. These interesting results can be explained by the opportunistic pathogenicity of both *E. coli* and *Candida* spp. [53,56].

When analyzing OPN distribution in our samples, we detected similar levels of this inflammatory and immune modulatory molecule in both the control samples and the samples showing Gram-negative enterobacteria. Our observations are similar to those of Salvi et al., who reported increased OPN levels in Gram-positive infections, which activate Toll-like receptor (TLR) 2, and lower levels in Gram-negative pathogens triggering TLR 3 and 4 [57]. Another interesting aspect of this pleiotropic molecule was revealed by Inoue et al.’s study in 2010 [58]. These authors describe two OPN isoforms: secreted OPN (sOPN) and intracellular OPN (iOPN), with the host’s resistance to fungal infection attributed to the iOPN isoform [58]. Despite the general trend to consider OPN as an inflammatory marker, with elevated levels during infections that modulate the host immune response, it appears that analyses of the involved mechanisms reveal more complex interactions than anticipated [57,58,59]. Our IHC analysis identified only secreted OPN; thus, this result may constitute a possible source of biases, but it also explains the low response to *Candida* spp. vaginal infection [60,61]. One of the most feared pathogens that succeed in employing a wide range of immune evasion strategies to elude the host’s defense mechanisms and to limit the host’s immune response is *Klebsiella*. The minimal activation of the immune response and its ability to evade detection are also extremely visible in the results we obtained [62]. This fearful micro-organism ranks last in our classification, displaying an extremely low positivity for VDR, CD44, OPN, and COX-2 compared to the control group.

The expression of all of the first three markers, except for COX-2, seems to correlate with the severity and the causative pathogens of vaginal infections in late pregnancy. Vitamin D supplementation might be extremely useful in lowering the host susceptibility to vaginal infections by lowering VDR placental expression. In addition, if our results are confirmed by larger population studies, we can attempt to develop local or systemic anti-CD44 and anti-OPN agents to try to control the severity of the host immune response during chorioamnionitis.

Study limitations: Our study limitations reside in the low number of included cases. Despite being aligned with the literature results, our findings need to be considered with caution until larger studies confirm them. Another limitation results from our inclusion of single-pathogen vaginitis, while many vaginal infections are caused by two or more pathogens. Additionally, our control group was composed of only women with term pregnancies. Another limitation is the lack of correlation with actual pathologies, but the results we obtained could form the basis for further research in this direction. We intend to expand our research and correlate our immunohistochemical findings with maternal and fetal outcomes.

## 4. Materials and Methods

### 4.1. Patients and Tissue Samples

The study period was from January 2021 to January 2023. All of the included patients provided written informed consent. This study was conducted in accordance with the Declaration of Helsinki and approved by the Ethics Committee of the University of Medicine and Pharmacy “Lucian Blaga”, Sibiu (1442/19 March 2024), and that of the Obstetrics and Gynecology Hospital “Cuza-Voda” in Iasi, Romania (10426/24 August 2021 and 19/4 August 2023).

#### 4.1.1. Inclusion Criteria

Specimens for the control group were collected from women who delivered at term without vaginal infections (both negative vaginal and cervical cultures), complications, associated diseases, or chronic treatment. These women also tested negative for acute infections (Toxoplasma, Rubella, Cytomegalovirus, Herpes, Human Immunodeficiency Virus, Syphilis, and Hepatitis B and C). For the vaginal infection group (positive vaginal/cervical cultures, more than 3+++ growth and significant inflammatory reaction), placental specimens were retrieved from age-matched women, with single pathogen-positive vaginal/cervical cultures, at >24 weeks of gestation, with PROM/PPROM, and with or without chorioamnionitis, that gave birth between 6 and 72 h after admission depending on their clinical and or paraclinical status (fever, leukocytosis, and elevated CRP levels), but were similarly free of other complications, associated diseases, or chronic treatment and negative for the above-mentioned acute infections before antibiotherapy initiation. In the vaginal infection group, we included only women with pregnancy over 24 weeks of gestation to avoid any other early placental alterations that might interfere with our observations. Stout et al. detected many placentas with intracellular bacterial colonization present in pregnancies under 24 weeks of gestation [32]. All of the included women were interviewed regarding their current medication, and those receiving antibiotic treatment were excluded from the study.

#### 4.1.2. Exclusion Criteria

Women with obstetrical and/or other medical complications were excluded from our analysis, including malignancy; depression; genetic syndromes; infectious or autoimmune disease; pre-existing or gestational diabetes; hypertension and its complications, such as pre-eclampsia; oligohydramnios; intrauterine growth restriction, defined as ultrasound-estimated fetal weight less than the 10th percentile for the gestational age; smoking habits; other therapies affecting bone and mineral metabolisms; or vitamin D supplementations within three months before pregnancy. We also excluded pregnant women with more than one pathogen detected in the vaginal culture in order to determine germ-specific placental molecular changes.

We used selective and differential culture media (MacConkey agar, Oxoid; Columbia agar and sheep blood, Oxoid; Sabouraud glucose selective agar with gentamicin and chloramphenicol, Oxoid) for the detection of vaginal infection pathogens. For bacterial identification, we utilized a MicroScan WalkAway analyzer manufactured by Beckman Coulter, Inc. (Brea, CA, USA), and for *Candida* spp. detection, an ELITech CANDIFAST^®^ kit (ELITECH MICROBIO, Signes, France) was used.

### 4.2. Immunohistochemistry

We collected four tissue samples from each placenta, one for each of the quadrants. The samples were collected from the maternal side of the placenta, aiming to harvest a sample from the edge, one from the center, and the two remaining samples at equal distances between the periphery and the center to ensure better coverage. The hematoxylin and eosin (H&E)-stained sections were examined by two independent pathologists with expertise in gynecological pathology. Differences were resolved through discussion and compromise until an agreement was reached. Immunohistochemistry (IHC) was performed and evaluated by two pathologists. IHC staining was performed on formalin-fixed, paraffin-embedded tissues, utilizing monoclonal antibodies against VDR, CD44, OPN, and COX-2. Four-micrometer-thick serial sections were prepared in citrate buffer (pH 6) after deparaffinization in xylene and rehydration in ethanol series. Endogenous peroxidase activity was inhibited with 0.3% H_2_O_2_ for 20 min at room temperature. IHC was performed to determine the expression of VDR, CD44, OPN, and anti-COX-2 using specific Abcam Company dilutions: for VDR, the dilution was 1:3000 (catalog no. ab3508, Abcam, Cambridge, UK); for CD44, it was 1:250 (catalog no. ab157107, Abcam, Cambridge, UK); for OPN, it was 1:200 (catalog no. ab8448, Abcam, Cambridge, UK); and for COX-2, it was 1:100 (catalog no. ab15191, Abcam, Cambridge, UK). The sections were incubated overnight at 4 °C. Then, the sections were washed, exposed to the secondary antibody for 45 min at 37 °C, and cleaned with phosphate-buffered saline (PBS). Hematoxylin was used as a counterstain with the standard avidin–biotin–peroxidase technique, using a liquid diaminobenzidine (DAB) substrate and a chromogen system. Human jejunum served as a positive control for VDR, CD44, OPN, and anti-COX-2 to ensure the specificity and sensitivity of the staining, validate the results, and rule out false positives or negatives. For the negative control, we treated the tissue samples in a way that should result in no staining, so the tissue samples would not express the antigen of interest.

All the placental samples were examined for the presence of VDR, CD44, OPN, and COX-2. Positive cells (brown or yellowish-brown color in the nucleus, cytoplasm, and plasma membrane) in the epithelial and stromal compartments were considered VDR-, CD44-, OPN-, and COX-2-positive, regardless of staining intensity or the number of positive cells. The expression levels of CD44, VDR, and COX-2 were quantified by assessing the percentage of positively stained cells and the staining intensity in each section. For overall positivity, immunostaining in >5% of cells was considered positive, and immunostaining in <5% positive cells was considered negative.

### 4.3. Statistical Analysis

The data were imported into Microsoft Excel and analyzed using SPSS 24 (IBM SPSS Statistics for Windows, Version 24.0, released in 2016, Armonk, NY, USA: IBM Corp.). Descriptive statistics included the sample size (absolute N and N% relative frequencies); mean; standard deviation; and 95% confidence intervals for the mean, quartiles, minimum, and maximum. Statistical hypothesis tests included nonparametric tests such as Mann–Whitney U test for 2-sample comparisons. These tests were applied for the analysis of continuous numerical variables. We used Chi-square test to detect statistical significance, with a standard significance level of 0.05 applied for making decisions, and we performed a univariate analysis.

## 5. Conclusions

We detected variability in the expression of molecules involved in placental adhesion, angiogenesis, migration, differentiation, and immune modulation (VDR, CD44, OPN, and COX-2) in different pathogenic vaginal infections. Gram-negative pathogens succeed in eluding the host immune system, and this aspect was also visible in our results, with lower levels of IHC positivity for the above-mentioned molecules in cases of vaginal infections with *E. coli*, *Enterobacter*, and *Klebsiella*. The pathogens with the highest IHC positivity for VDR, CD44, OPN, and COX-2 were *GBS* and *Ureaplasma urealyticum*. In addition, the most frequently observed vaginal infection during pregnancy was due to *Candida* spp., which resulted in low IHC expression of these molecules.

This study evaluated the differential expression of placental markers in response to pathogens, which can contribute to our understanding of pathogen-specific impacts on pregnancy outcomes. Further research is needed to directly correlate the changes in these markers with clinical pathologies. These insights underscore the potential for targeted therapeutic interventions aimed at modulating these pathways to improve pregnancy outcomes.

## Figures and Tables

**Table 1:** Clinical and demographic characteristics of the included women.

		Mean	St. Dev.	95.0% CI for Mean		Percentile 25	Median	Percentile 75	Min	Max	Mann– Whitney U *p*-Value
				Lower Bound	Upper Bound						
Age (years)	Control group	27.8	4.3	26.8	28.8	27	29	31	19	34	0.13
	Vaginal infection group	29.3	6.3	27.8	30.7	25	30	34	17	43	
Weeks of gestation	Control group	39.7	1.4	39.4	40	38	40	41	37	41	<0.01
	Vaginal infection group	34.6	5.3	33.4	35.8	30	36	39	24	41	
Fetal birth weight (g)	Control group	3503.1	313.9	3428.3	3578	3360	3530	3670	2800	4340	<0.01
	Vaginal infection group	2557.3	1160	2295.7	2818.9	1350	2920	3520	570	4540	
APGAR score	Control group	8.5	0.5	8.4	8.6	8	9	9	8	9	0.28
	Vaginal infection group	8.1	1.5	7.7	8.4	7	8	9	4	10	
Antepartum hemoglobin level (milligrams/deciliter)	Control group	12.3	1.1	12.0	12.6	11.8	12.1	13.6	10.0	13.7	0.34
	Vaginal infection group	12.0	1.1	11.8	12.3	11.4	12.2	12.7	9.5	14.3	
Postpartum hemoglobin level (milligrams/deciliter)	Control group	10.9	.8	10.8	11.1	10.4	10.9	11.5	9.0	12.1	0.26
	Vaginal infection group	11.1	3.2	10.4	11.8	10.0	10.8	11.4	9.0	37.3	
Antepartum hematocrit level (%)	Control group	36.3	3.4	35.5	37.1	35.0	37.0	38.9	28.1	40.0	0.15
	Vaginal infection group	35.2	5.2	34.0	36.4	34.3	36.1	38.3	12.2	42.1	
Postpartum hematocrit level (%)	Control group	31.8	2.9	31.1	32.5	29.0	31.1	34.3	27.8	37.0	0.41
	Vaginal infection group	31.3	3.3	30.5	32.0	28.5	30.4	33.6	25.6	38.5	
Leucocyte count (10^3^/L)	Control group	13,184	3874	12,311	14,058	10,260	12,970	16,110	7040	25,240	0.89
	Vaginal infection group	25,920	41,820	15,949	35,892	10,350	11,485	15,430	9900	150,303	
Platelet count (10^6^/L)	Control group	220,171	78,308	201,500	238,843	147,000	217,500	278,000	132,000	355,000	0.46
	Vaginal infection group	225,705	66,226	210,773	240,637	170,000	218,000	260,000	123,000	362,000	
C reactive protein value (milligrams/deciliter)	Control group	3.12	1.52	2.76	3.49	2.00	2.75	4.10	1.00	10.00	<0.01
	Vaginal infection group	24.92	26.61	18.92	30.92	4.50	12.98	36.00	1.90	98.00	

**Table 2:** VDR, CD44, OPN, and anti-COX-2 placental expression in vaginal infections during pregnancy.

		VDR		CD44		OPN		COX-2		Total
		Negative	Positive	Negative	Positive	Negative	Positive	Negative	Positive	
*Candida* spp.	Count	2	10	10	2	5	7	1	11	12
	% within cultura	16.7%	83.3%	83.3%	16.7%	41.7%	58.3%	8.3%	91.7%	100.0%
*Escherichia coli*	Count	3	13	0	16	16	0	0	16	16
	% within cultura	18.8%	81.3%	0.0%	100.0%	100.0%	0.0%	0.0%	100.0%	100.0%
*Enterococcus faecalis*	Count	1	13	2	11	11	2	0	13	13
	% within cultura	7.1%	92.9%	15.4%	84.6%	84.6%	15.4%	0.0%	100.0%	100.0%
*Klebsiella*	Count	9	1	9	1	8	2	0	10	10
	% within cultura	90.0%	10.0%	90.0%	10.0%	80.0%	20.0%	0.0%	100.0%	100.0%
*Streptococcus beta hemolitic*	Count	1	13	1	14	2	13	0	15	15
	% within cultura	7.1%	92.9%	6.7%	93.3%	13.3%	86.7%	0.0%	100.0%	100.0%
*Ureaplasma urealyticum*	Count	1	11	1	11	3	9	0	12	12
	% within cultura	8.3%	91.7%	8.3%	91.7%	25.0%	75.0%	0.0%	100.0%	100.0%
Total	Count	17	61	23	55	45	33	1	77	78
	% within cultura	24.6%	75.4%	29.5%	70.5%	57.7%	42.3%	1.3%	98.7%	100%

**Table 3:** VDR, CD44, OPN, and anti-COX-2 placental expression in the vaginal infection group.

			VDR		CD44		OPN		COX-2		Total
			Negative	Positive	Negative	Positive	Negative	Positive	Negative	Positive	
Group	Vaginal infections	Count	16	62	23	55	45	33	1	77	78
		%within lot	20.51%	79.49%	29.5%	70.5%	57.7%	42.3%	1.3%	98.7%	100%
	No vaginal infections	Count	14	56	14	56	56	14	0	70	70
		%within lot	20%	80%	20%	80%	80%	20%	0%	100%	100%
Total		Count	30	118	37	111	101	47	1	147	148
		%within lot	20.27%	79.7%	25%	75%	68.2%	31.8%	0.7%	99.3%	100%

**Table 4:** Statistical significance of the analyzed markers.

Analyzed Markers	VDR	CD44	OPN	COX-2
Sig.	0.907	0.185	0.004	Not computed
Exp (B)	0.953	0.598	2.933	
Lower	0.427	0.279	1.402	
Upper	2.129	1.280	6.136	
OR	1.102	1.67	2.933	

## Data Availability

The data used to support the findings of this study are available upon request to the corresponding author.
